# Two superoxide dismutases from Tn*Otchr* are involved in detoxification of reactive oxygen species induced by chromate

**DOI:** 10.1186/s12866-016-0648-0

**Published:** 2016-03-05

**Authors:** Rita Branco, Paula V. Morais

**Affiliations:** CEMUC-Department of Mechanical Engineering, University of Coimbra, 3030-788 Coimbra, Portugal; Department of Life Sciences, University of Coimbra, 3001-401 Coimbra, Portugal

**Keywords:** Superoxide dismutases, Reactive oxygen species, Chromate stress, Mutant cells, Fluorescent dyes

## Abstract

**Background:**

Superoxide dismutases (SOD) have been reported as the most relevant bacterial enzymes involved in cells protection from reactive oxygen species (ROS). These toxic species are often the product of heavy metal stress.

**Results:**

Two genes, *chrC* and *chrF*, from Tn*Otchr* genetic determinant of strain *Ochrobactrum tritici* 5bvl1 were cloned in *Escherichia coli* in order to overexpress the respective proteins. Both proteins were purified and characterized as superoxide dismutases. ChrC was confirmed as being a Fe-SOD, and the enzymatic activity of the ChrF, not inhibited by hydrogen peroxide or potassium cyanide, suggested its inclusion in the Mn-SOD family. This identification was supported by chemical quantification of total metal content in purified enzyme. Both enzymes showed a maximum activity between pH 7.2-7.5. ChrF retained nearly full activity over a broader range of pH and was slightly more thermostable than ChrC. The genes encoding these enzymes in strain *O. tritici* 5bvl1 were inactivated, developing single and double mutants, to understand the contribution of these enzymes in detoxification mechanism of reactive oxygen species induced by chromate. During chromate stress, assays using fluorescent dyes indicated an increase of these toxic compounds in *chrC*, *chrF* and *chrC/chrF* mutant cells.

**Conclusions:**

In spite of the multiple genes coding for putative superoxide dismutase enzymes detected in the genome of *O. tritici* 5bvl1, the ChrC and ChrF might help the strain to decrease the levels of reactive oxygen species in cells.

## Background

Oxygen present in the environment is potentially toxic to organisms because of the toxicity of reactive oxygen species (ROS) that are generated as by-products during the reduction of oxygen to water [1]. ROS such as superoxide radicals (O_2_^.−^), hydrogen peroxide (H_2_O_2_) and hydroxyl radical (^∙^OH) enforce oxidative damage to the cells, for instance DNA strand breakage, protein inactivation, and membrane lipid peroxidation [2]. In order to deal with oxidative stress and to avoid the harmful effects of the ROS, most organisms have developed additional defense systems, which include superoxide dismutases (SODs) [3]. SODs are enzymes involved in the detoxification of O_2_^.-^ to H_2_O_2_ catalyzing the reaction: 2O_2_^.-^ + 2H^+^ → H_2_O_2_ + O_2_. Then, H_2_O_2_ is broken down to water by catalases and peroxidases [3, 4]. Therefore, SODs play a vital role in the primary defense line mechanisms against the oxidative stress.

SODs are metalloenzymes that can be classified into four groups according to their metal cofactor: the iron SOD (Fe-SOD), the manganese SOD (Mn-SOD), the copper zinc SOD (CuZn-SOD), the nickel SOD (Ni-SOD) [5]. There is also a particular group of SODs, the cambialistic SOD, that can function well either with iron or manganese at its active site [6, 7]. The Cu/Zn-SODs are predominantly found in eukaryotes and in a few bacteria [8]. Most bacteria contain Fe- and/or Mn-SODs and some Ni-SODs have also been discovered in several *Streptomyces* species [9].

The expression of SODs in microorganisms is often related with response to heavy metal stress [10–12]. For instance, *Rhodobacter capsulatus* cells incubated with tellurite exhibited an increase in superoxide dismutase activity [13]. Proteome analysis of selenite response of *Rhodobacter sphaeroides* also showed enhanced synthesis of enzymes associated to oxidative stress [12].

It is well documented that ROS are products of Cr(VI) reduction and microorganisms in chromium contaminated environments should have developed defense systems against oxidative stress [14]. Bacterial cells when exposed to chromate activate several protective systems, including superoxide dismutase and catalase enzymes [15, 16]. In our previous work, we have studied the genetic organization of a chromate resistance determinant (Tn*Otchr*) of a highly resistant strain, *Ochrobactrum tritici* 5bvl1 [17]. That work identified a set of Cr(VI) resistance genes: *chrB* encoding a chromate regulator [18], *chrA* encoding a chromate transporter and *chrC* and *chrF*, two SOD-like genes. ChrC of strain 5bvl1 shows similarity to an identified Fe-SOD from *Cupriavidus metallidurans* [19]. In a previous work, ChrF and ChrC did not seem to play a crucial role in chromate resistance but, their expression in *Escherichia coli* increased resistance of cells to toxicity of reagents generating superoxide anions (17). However, little is known about specific features of ChrC and, up to date, there is no characterization of ChrF.

In the present work, these two putative SODs were characterized and their metal cofactors, sensitivity to inhibitors and molecular properties (molecular weight and protein oligomerization) were determined. Cloning and expression of the *chrC* and *chrF* genes in *E. coli* was performed in order to investigate the biochemical properties of the enzymes. Moreover, with the construction of *chrC* or/and *chrF O. tritici* mutants, the importance of these enzymes in the process of intracellular detoxification of ROS, generated by chromate, was demonstrated.

## Methods

### Bacterial strains, plasmids and growth conditions

Bacterial strains and plasmids used in this study are shown in Table 1. *O. tritici* 5bvl1 strains were grown aerobically at 35 °C in Luria-Bertani (LB) medium containing 10 g/L tryptone, 5 g/L yeast extract and 5 g/L NaCl. When required, this medium was supplemented with ampicillin (100 μg/mL), kanamycin (50 μg/mL) and sucrose (5 %). The strain *E. coli* BL21(DE3) was used for the expression of *chrC* and *chrF* and strain *E. coli* S17-1 was used as donor cells during the conjugation procedure.

**Table 1:** Bacterial strains, plasmids and primers used in this work

Strain or Plasmid	Relevant Characteristic(s)	Reference or Source
Bacterial Strains		
*O. tritici* 5bvl1	Type strain; Amp^r^; Cr(VI)^r^	
*chrC* mutant	Single mutant of 5bvl1; *chrC* mutated	This study
*chrF* mutant	Single mutant of 5bvl1; *chrF* mutated	This study
*chrC/chrF* mutant	Double mutant of 5bvl1; *chrC* and *chrF* mutated	This study
*E. coli* S17-1	Conjugation donor strain	22
*E. coli* 21(DE3)	F^−^ *ompT hsdS* (r_B_^-^, m_B_^−^) *gal dcm lacY1*(DE3)	Novagen
Plasmids		
pJQ200SK	Suicide vector; *sacB*; Gm^r^	ATCC
pET30a	Kmr, expression plasmid	Novagen
petChrC	pET30a for overproduction of ChrC with an C-terminal hexahistidine tag	This study
petChrF	pET30a for overproduction of ChrF with an C-terminal hexahistidine tag	This study
p*chrC*	pJQ200SK derivative carrying the upstream and downstream regions of chrC gene	This study
Primers		
NdechrCf	ATA**CATATG**TCCTTCGACATTAAACCGC	This study
SalchrCr	TCC**GTCGAC**TGAGAGTCCTCCTGAGGGTTG	This study
NdechrFf	CGA**CATATG**AAATGGATTACCCGCGAAC	This study
SalchrFr	CGA**GTCGAC**CATCTTCGGCGGCCAGTTATG	This study
chrCupf	GCT**GGATCC**TCTGGCTTGCTGCGGGTTGCC	This study
chrCupr	GCT**CTGCAG**TCACAAGATCAAGGTCTTCAA	This study
chrCdownf	GCT**CTGCAG**ATGAAATGGATTACCCGCGAA	This study
chrCdownr	CGA**CTCGAG**TCACATCTTCGGCGGCCAGTT	This study
chrFupf	GCT**GGATCC**AACGGCGTGTTGCCCGTAGGC	This study
chrFupr	GCT**CTGCAG**TCATGAGAGTCCTCCTGAGGG	This study
chrF’f	GCT**CTGCAG**CCATGACGGTGAGCTTTGCAG	This study

Enzyme restriction site in primer sequences is shown in bold

### Expression and purification of SODs

The full-length of *chrC* and *chrF* genes were amplified from *O. tritici* 5bvl1 genomic DNA using the primer pairs NdechrCf/SalchrCr and NdechrFf/SalchrFr, respectively (Table 1) that corresponded to regions of their open-reading frames. The stop codon of each gene was removed from the reverse primer to allow the translation of a C-terminal His6-tag encoded by the expression vector pET30a (Novagen, San Diego, CA). The PCR products were digested with the respective restriction enzymes, electrophoresed and extracted from the gel. Then, DNA fragments were ligated into a pET30a vector, resulting in petChrC and petChrF plasmids, and transformed into competent *E. coli* BL21(DE3). The cloned genes were verified by DNA sequencing. Both proteins, ChrC-His6 and ChrF-His6 were overexpressed and purified using the same strategy. *E. coli* BL21(DE3) carrying the plasmids petChrC or petChrF were grown overnight at 37 °C in LB containing kanamycin. The cultures were diluted 1:10 into 1 L of LB with kanamycin and incubated at 37 °C until 0.5 of optical density (OD) at 600 nm. Then, isopropyl-D-thiogalactopyranoside (IPTG) was added to a final concentration of 0.5 mM, and incubation was continued overnight at 25 °C. Bacterial cells were harvested, resuspended in 20 mM sodium phosphate buffer at pH 7.4 with 0.5 M NaCl and 20 mM imidazole. A protease inhibitor cocktail (Roche, Mannheim, Germany), 10 μg/mL DNAse I and 5 mM MgCl_2_ were added to the suspension. Cells were disrupted twice in a French-press cell followed by centrifugation (15000 × *g*, 4 °C, 40 min). The recombinant ChrC and ChrF proteins were purified in a prepacked Ni-Sepharose high-performance column (His-Prep FF 16/10) equilibrated with 20 mM sodium phosphate, pH 7.4, 0.5 M NaCl, and 20 mM imidazole. Elution was carried out with 500 mM imidazole and the eluted fractions containing the majority of ChrC or ChrF were concentrated by centrifugation in 10 kDa cutoff centricons (Millipore, Bedford, MA), equilibrated with 50 mM Tris, pH 7.4. The purity of fractions was assessed by electrophoresis on a 0.1 % sodium dodecyl sulfate (SDS)-12 % polyacrylamide gel, followed by Coomassie blue staining. The purified ChrC-His6 and ChrF-His6 proteins were stored in buffer Tris 50 mM, pH 7.4 at 4 °C. Protein concentrations were determined by using the Bradford assay (Bio-Rad, Hercules, CA) and bovine serum albumin (BSA) (Sigma, St. Louis, MO) as the protein standard.

### SOD activity staining

SOD activity was visualized on a non-denaturing polyacrylamide gel as previously described [20]. Proteins (10 μg) were subjected to 10 % native-PAGE. Gels were incubated with 0.1 % Nitroblue tetrazolium (NBT) solution in dark with shaking for 15 min at room temperature and then incubated with Riboflavin solution (28 μM riboflavin and 28 mM TEMED in 0.1 M potassium phosphate buffer, pH 7.0) in dark with shaking for 15 min at room temperature. Gels were illuminated with a white-light box at room temperature and the SOD activity area appeared as a clear zone on a blue-violet background. The effect of several compounds on enzymes activity was tested to differentiate both SODs. The enzyme (10 μg) was mixed with potassium cyanide (KCN, 10 mM), sodium azide (NaN_3_, 10 mM) or hydrogen peroxide (H_2_O_2_, 10 mM) and incubated at 30 °C for 1 h. After incubation, the SOD activity was assayed in native gels as describe above.

### SOD assay in solution

SOD activity was determined using the photochemical microplate assay method [21] and measuring enzyme ability to inhibit the photochemical reduction of NBT. The reactions were performed in 50 mM phosphate buffer pH 7.5 or 50 mM Tris–HCl buffer pH 7.2, for ChrC or ChrF assays, respectively. Besides the buffer solutions, the reaction mixture was composed by 13 mM methionine, 75 μM NBT, 2 μM riboflavin, 0.1 mM EDTA, and 2 μg of enzyme. Riboflavin was added last into the reaction mixture. The microplate was placed 30 cm below two 40-W lamps and the reaction was run for 15 min. Absorbance was read at 560 nm using a spectrophotometer (Infinite M200, Fisher). Reaction mixture without enzyme was also performed as a control, which developed the maximum color.

In cellular extracts, the total SOD activities were defined as U/mg protein and one enzyme unit corresponds to 50 % inhibition of the reaction.

### Optimal pH, thermal and pH stability assays

To determine the optimal pH for SOD activity, the purified enzymes were assayed in 50 mM of several buffers instead of the assay solution described above. The buffer solutions used were 50 mM citrate buffer (pH 4–6), potassium phosphate buffer (pH 6–8), Tris–HCl Buffer (pH 7.2–10) and carbonate-bicarbonate buffer (pH 11). After 15-min incubation at 30 cm below two 40-W lamps, SODs activities were measured.

The pH stability of ChrC and ChrF enzymes were tested by incubation in solutions ranging from pH 4 to 11 for 3 h. The thermal stability of ChrC and ChrF was determined by incubating the purified enzymes at temperatures from 22 to 65 °C for 3 h. After incubation time, the remaining SOD activity was determined, as indicated above under standard conditions and calculated as the percentage of the maximum SOD activity.

### Metal analyses

Metal present in the purified ChrC and ChrF proteins were analyzed using Inductively Coupled Plasma Mass Spectrometry (ICP-MS) in an ICP-MS Thermo X Series. First, protein was frozen at −20 °C for 10 min, heated at 50 °C for 60 to 120 min and then centrifuged at 4000 rpm for 30 min, at 4 °C. The supernatants were collected and submitted to analyses of iron, manganese, nickel, zinc and copper.

### Evaluation of the oligomeric state of ChrC and ChrF

To determine whether ChrC and ChrF are in oligomeric form, chemical crosslinking assays were performed using glutaraldehyde. Reaction mixtures containing 10 μg of purified enzymes in crosslinking buffer (20 mM NaCl, 10 mM KCl, 2 mM DTT in 20 mM Hepes, pH 7.5) were incubated with glutaraldehyde to a final concentration of 0.1 %, and the reaction mixture was incubated for 5, 15 and 30 min at 30 °C. Crosslinking was terminated by adding SDS-PAGE sample buffer, heating at 95 °C for 5 min, and the samples analyzed by 12 % SDS-PAGE.

The purified ChrC and ChrF were subjected to gel filtration using a Superdex 200 10/300 GL packed column (GE Healthcare). The elution volume of standard proteins of alcohol dehydrogenase (150 kDa), bovine serum albumin (BSA) (66 kDa), carbonic anhydrase (29 kDa), and ribonuclease (13.7 kDa) were first detected, followed by gel filtration of the recombinant enzymes under the same conditions. The molecular mass curve of the standard proteins was thus constructed and the molecular mass of ChrC and ChrF was calculated.

### Construction of *Ochrobactrum tritici* 5bvl1 mutants

Single *chrC* mutant was constructed by deletion of the *chrC* gene from *chr* operon of native strain. Briefly, the upstream gene portion of *chrC* gene, of around 400 bp, amplified by specific primers, chrCupf and chrCupr and the downstream gene portion of *chrC* gene, of about 450 bp, amplified using the specific primers, chrCdownf and chrCdownr were digested with the pair of enzymes respectively. These fragments were cloned into pJQ200sk vector at the *Bam*HI/*Pst*I and *Pst*I/*Xho*I restriction sites resulting in p*chrC* plasmid. This plasmid was transformed into *E. coli* S17-1 and transferred to the recipient strain *O. tritici* 5bvl1 by biparental conjugation using the filter mating method [22]. Double-crossover transconjugants were selected on LB plates with ampicillin and sucrose. Positive mutants (*chrC* mutant) were confirmed by PCR using the specific primers to amplify the *chrC* gene. Single mutant, *chrF* mutant, was obtained by removing part of the *chrF* gene of strain 5bvl1. Succinctly, a fragment of about 300 bp corresponding to the upstream gene portion of *chrF* gene was amplified by specific primers, chrFupf and chrFupr. The terminal *chrF* gene portion of 280 bp was amplified using the specific primers chrF’f and SalchrFr. As above, the PCR products were digested with the pair of enzymes respectively, cloned into pJQ200sk vector, transformed into *E. coli* S17-1 and transferred to the strain 5bvl1. The transconjugants were also selected on LB plates with ampicillin and sucrose. Positive mutants (*chrF* mutant) were confirmed by PCR using the specific primers to amplify the total *chrF* gene. Double mutant, *chrC/chrF* mutant, was constructed using the previous strategies to delete the *chrC* gene and remove partially the *chrF* gene. Thus, the suicide plasmid p*chrC* transformed in *E. coli* S17-1 was used to conjugate with the recipient strain *chrF* mutant. Transconjugants were selected on LB plates with ampicillin and sucrose and positive clones (*chrC/chrF* mutant) were confirmed by PCR.

### Determination of intracellular oxidation levels

The oxidant-sensitive probe 2',7'-dichlorodihydrofluorescein diacetate (H_2_DCFDA, Sigma Aldrich, Germany) [23] was used to determine the intracellular levels of ROS in *O. tritici* 5bvl1 wild type and mutant cells untreated and treated with chromate. Cells were grown overnight aerobically in LB medium or in chromate (0,5; 1; 2 mM)-amended LB medium. Then, cells were diluted at OD 0.5 and washed twice with Phosphate Buffer Salt (PBS) (pH 7.0). Cells were incubated for 30 min in the same buffer containing 25 μM H_2_DCFDA dissolved in dimethyl sulfoxide. After three washing steps, the cells were suspended in the same buffer and the fluorescence intensity was immediately measured at each 1 h interval, over a 3 h period, using fluorescence microplate reader (Infinite M200, Fisher) (excitation, 495 nm; emission, 517 nm). All the values were normalized by optical density measured at 600 nm.

## Results

### Expression and molecular properties of enzymes

**Fig. 1:**
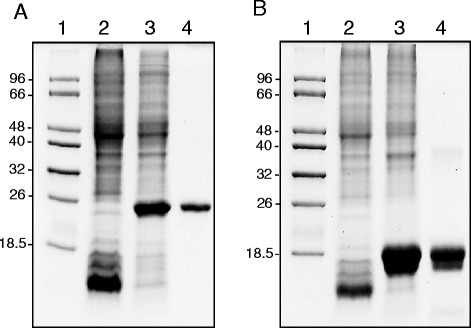
ChrC (**a**) and ChrF (**b**) were overexpressed with carboxy-terminal 6-histidine tags in *E. coli* BL21 (DE3). Lane 1, DNA ladder; lane 2, whole-cell extract from uninduced cells containing pET30a expressing the proteins; lane 2, whole-cell extract from induced cells containing pET30a expressing the proteins; lane 3, purified ChrC-His6 (**a**) and ChrF-His6 (**b**) by Ni2+ nitrilotriacetic acid affinity chromatography

The genes *chrC* and *chrF* of Tn*Otchr* of *O. tritici* 5bvl1 code for two putative SODs with different subcellular localization predicted by the informatics program PSORTb version 3.0.2 [24]. While ChrC was identified as a putative periplasmic protein (score 9.44), ChrF was identified as a cytoplasmic protein (score 8.96). In this work, the genes *chrC* and *chrF* of *O. tritici* 5bvl1 were highly expressed in *E. coli* BL21(DE3). The heterologous proteins were purified using Ni-NTA affinity chromatography. The monomeric molecular mass of the recombinant ChrC and ChrF was found to be approximately 25 kDa and 19 kDa, respectively (Fig. 1). These masses corresponded to the sum of each predicted molecular mass of the proteins (~22 kDa and ~17 kDa) and the (His)6 tag (~2.6 kDa).

**Fig. 2:**
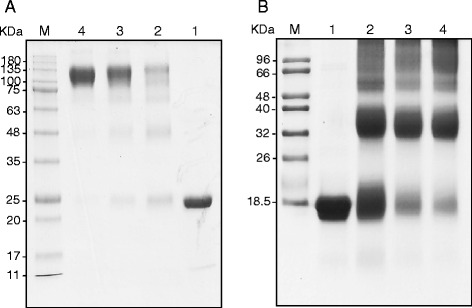
Chemical crosslinking assays. SDS-PAGE of purified ChrC (**a**) and ChrF (**b**) in glutaraldehyde crosslinking assays. Lane 1, protein without glutaraldehyde; lane 2, protein + glutaraldehyde (5 min); lane 3, protein + glutaraldehyde (15 min); lane 4, protein + glutaraldehyde (30 min)

To determine the oligomeric status of both proteins, chemical crosslinking experiments were performed on purified recombinant enzymes. Analysis of the reaction mixtures that contained purified ChrC-His6 and ChrF-His6 with glutaraldehyde resulted in shifting position bands on SDS-PAGE. Increasing incubation time of ChrC with glutaraldehyde resulted in the appearance of a strong band on the gel slightly above of 100 kDa, corresponding to a tetramer (Fig. 2a). Incubation of ChrF with glutaraldehyde over time caused a shifting of the monomeric to dimeric status of protein, revealed by appearance of a strong band at position of approximately of 35 kDa on gel (Fig. 2b).

**Fig. 3:**
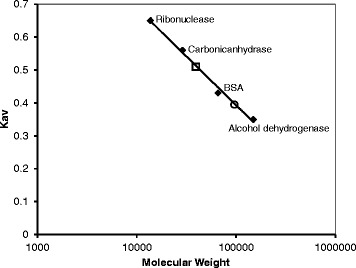
Determination of the molecular mass of ChrC and ChrF by gel filtration. Chromatography was performed on Superdex 200 10/300 GL packed column at a flow rate of 0.5 mL/min with 50 mM Tris–HCl, 0.15 M NaCl, pH 7.4. K_av_ values were calculated using the equation: K_av_ = (Ve-Vo)/(Vc-Vo), where Vo is column void volume, Ve is elution volume, and Vc is geometric column volume. The studied proteins are represented by open symbols: ChrC (○); ChrF (□)

The molecular mass of the recombinant ChrC and ChrF proteins was also analyzed by size exclusion chromatography and determined as 96.2 kDa and 39.4 kDa, respectively, (Fig. 3). These results suggest that ChrC is a tetrameric protein comprising four subunits of 24.6 kDa and ChrF is a dimer comprising two subunits of 19.6 kDa. These molecular masses were in agreement with the results obtained from the chemical crosslinking assays.

### Identification of the metal cofactor

**Fig. 4:**
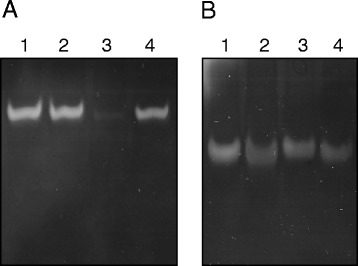
Activity staining of the purified ChrC (**a**) or ChrF (**b**) proteins electrophoresed on 10 % nondenatured polyacrylamide gel. Lane 1, enzymatic reaction in absence of inhibitor; lane 2, enzyme incubated with 10 mM KCN; lane 3, enzyme incubated with 10 mM H_2_O_2_; lane 4, enzyme incubated with 5 mM NaN_3_

The activity of ChrC and ChrF as SODs were evaluated on non-denaturing polyacrylamide gels. The purified recombinant proteins showed achromatic zones in activity staining gels revealing that both enzymes exhibit activity of SOD (Fig. 4). Additionally, compounds, such as H_2_O_2_, NaN_3_ and KCN were tested to assess their inhibitory effect on the activities of both enzymes. Figure 4a shows no white band when ChrC was incubated with H_2_O_2_, indicating that ChrC was inhibited by H_2_O_2_ and not by KCN, an expected characteristic of the Fe-SODs. On the other hand, there was no difference between the bands obtained on activity gels when ChrF was treated with the inhibitors and the control (Fig. 4b). These results indicate that ChrF activity was not inhibited by cyanide or H_2_O_2_, a common characteristic of Mn-SODs. Both enzymes were not inhibited by azide in the tested concentration.

The metal cofactors of both proteins were also confirmed through quantification of the metal content by ICP-MS. These analyses showed that iron was the most abundant metal in the purified ChrC protein (1.4 mol Fe/mol enzyme) and manganese, with 1.0 molMn/mol enzyme, was the major element present in the ChrF protein sample (Table 2). Therefore, the results obtained by inhibition tests and metal analyses are coherent indicating that ChrC and ChrF are a Fe-SOD and Mn-SOD, respectively.

**Table 2:** Metal contents of ChrC and ChrF

Metal	mol metal/mol of ChrC	mol metal/mol of ChrF
Cu	0.003	0.013
Fe	1.4	0.073
Mn	0.32	1.0
Ni	0.043	0.028
Zn	0.07	0.11

### Effect of metals on SODs activity

**Fig. 5:**
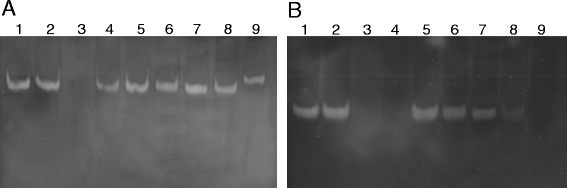
Effect of several metal ions on SOD activity of purified ChrC (**a**) or ChrF (**b**) enzymes. Lane1, enzymatic reaction in absence of any tested compound; lane 2, enzyme incubated with chromate, lane 3, enzyme incubated with Cr(III); lane 4, enzyme incubated with Fe(II); lane 5, enzyme incubated with Mn(II); lane 6, enzyme incubated with Ni(II); lane 7, enzyme incubated with Zn(II); lane 8, enzyme incubated with Cd(II); lane 9, enzyme incubated with Cu(II)

The assays of ChrC and ChrF with several metals showed distinct effects of metals on the SODs activity revealed by native staining gels. The protein ChrC was only inhibited by Cr(III) (Fig. 5a). On the other hand, ChrF was completely inhibited by Cr(III), Fe(II) and Cd(II) and was partially inhibited by Cu(II) (Fig. 5b).

### Optimum pH, stability to pH and temperature

**Fig. 6:**
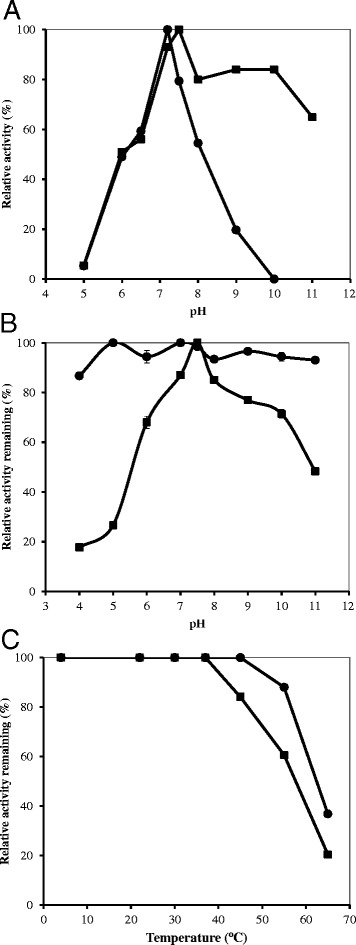
Effect of pH on SODs activity (**a**) and stability of SODs to pH (**b**) and temperature (**c**). **a** The purified enzymes were assayed at different pHs. **b** pH stability was determined by incubating the proteins in different pHs for 2 h prior to SOD assays at their optimum pH. **b** Thermal stability was determined after incubation of the purified proteins for 2 h at the indicated temperatures. ChrC assays (■); ChrF assays (●)

The effect of pH on the activity of purified SOD enzymes was determined using buffers at various pH values. The ChrC enzyme was highly active over a broad range of pHs tested (6.0 - 11.0) and showed maximum activity in phosphate buffer pH 7.5. ChrF was optimally active at Tris–HCl pH 7.2 and showed activity over a narrower range of pHs (6.0 - 9.0) (Fig. 6a).

For the pH stability test, enzymes were incubated for 3 h at various pHs and then assayed at the optimum pHs. The ChrF retained nearly full activity over a broad range of pH (4.0–11.0) and the ChrC held more than 50 % of total activity at pH between 5.0 – 10.0 but its stability was seriously affected at low pHs (Fig. 6b).

Both enzymes showed high thermal stability at temperatures lower than 45 °C, retaining more than 80 % of their activities (Fig. 6c) and both were unstable at temperatures above 65 °C. Moreover, over all tested temperatures, ChrF was slightly more stable than ChrC.

### Effect of chromate on the generation of ROS in *O. tritici* 5bvl1

**Fig. 7:**
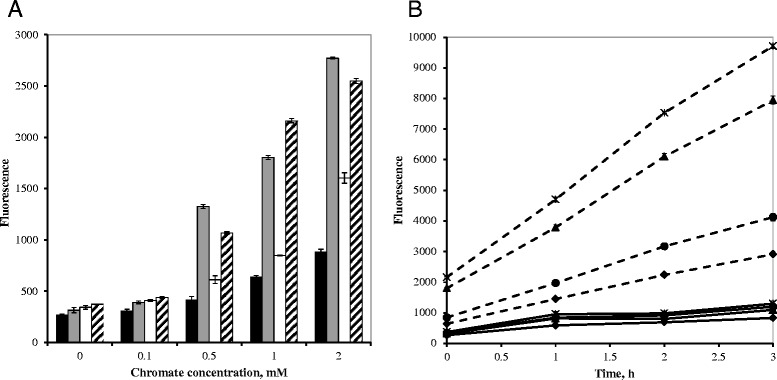
Generation of intracellular ROS by chromate. **a** Cytoplasmic ROS content was evaluated measuring the H_2_DCFDA probe activation in cells of, wild strain *O. tritici* 5bvl1 (*black bar*); *chrC* mutant (*grey bar*); *chrF* mutant (*white bar*) and *chrC/chrF* mutant (*dashed bar*), treated with indicated concentrations of chromate. **b** Comparative fluorescence signals of cells untreated (*solid line*) or treated (*dashed line*) with 1 mM chromate over time for wild strain *O. tritici* 5bvl1 (♦); *chrC* mutant (▲); *chrF* mutant (●) and *chrC/chrF* mutant (x)

To investigate whether ROS is involved in the toxicity of chromate, the intracellular ROS levels of control and chromate-exposed cultures were compared. When the results obtained immediately after the 30 min of incubation of cells with the fluorescent probe were compared, we observed that wild cells showed only a slight increase of fluorescence level at concentrations of chromate upper than 1 mM. On the other hand, the mutated cells, mostly the *chrC* and *chrC/chrF* mutants, showed a dramatic high level of fluorescence when exposed to chromate concentration over 0.5 mM (Fig. 7a). Interestingly, the fluorescence signal was not proportional to chromate concentration, implying that 0.5 mM of chromate was enough to induce high ROS generation in mutated cells. Figure 7b shows the fluorescence measured over time after the probe removal. It is noticeable that, in the case of cells not exposed to chromate, fluorescence did not increase. However, all cells exposed to 1 mM of chromate showed continuously increase of fluorescence level. Moreover, the intensification of fluorescence signal was stronger for *chrC* and *chrC/chrF* mutants.

Measurement of total superoxide dismutase activities upon chromate exposure was also analyzed for wild-type strain and mutants. The mean values of SOD activity of the wild type was higher (780 ± 193 U/mg protein) than mutants (622 ± 189 U/mg protein of *chrC*, 622 ± 191 U/mg protein of *chrF* and 600 ± 210 U/mg protein of *chrC/chrF*). Statistical analysis of all data (8 independent assays) performed by two-way ANOVA with Tukey´s multiple comparison post-test showed significant difference among mutants and control cells (*P* < 0.05).

## Discussion

Bacterial chromate resistance is often associated to the presence of a *chr* genetic determinant carrying, at least, a *chrA* gene coding for a well-known chromate efflux pump [14]. However, in many strains, the *chr* operon is also composed by others genes coding for proteins not sufficiently studied or characterized. In this work, ChrC and ChrF were characterized and their features compared since the respective genes, *chrC* and *chrF*, belong to the chromate inducible operon Tn*Otchr* [17]. The results from this study identified ChrC and ChrF as two different SODs, a Fe-SOD and Mn-SOD respectively. In fact, the strong sequence homology between ChrCs of *O. tritici* and *C. metallidurans* has already suggested that enzyme was a Fe-SOD [19]. This enzyme exhibited typical FeSOD-like characteristics in sensitivity to inhibitors i.e. it was inactivated by H_2_O_2_ [25, 26]. On the other hand, ChrF was seen for the first time as an enzyme with SOD activity. Its insensitivity to the tested inhibitors, H_2_O_2_; KCN, NaN_3_, suggested that ChrF in *O. tritici* 5bvl1 may be a member of the Mn-SODs. This was supported by the presence of 1.0 mol of Mn per mole of purified enzyme. In general, Fe-SODs are the most well characterized SODs but more recently, the biological functions of Mn-SOD have deserved a special attention by researchers. This type of enzyme is reported to be involved in different processes such as senescence, cell impairment and carcinogenesis [27, 28].

Besides the different sensitivity of both SODs to the inhibitors, these enzymes also exhibited others distinct features. In pH stability test, the enzyme ChrF was remarkably stable, retaining nearly full activity between pH 4–11. The different oligomerization between these two SODs (ChrC – tetramer; ChrF - dimer) could possibly explain the different pH stability. The susceptibility of ChrC, mainly to acidic pHs, may be due to the reported effect of acidic conditions in the dissociation of the functional tetramers into monomers, disturbing the enzyme activity [21]. Although, both enzymes were thermostable up to 45 °C retaining more than 80 % of SOD activity, ChrF showed more thermal stability than ChrC. In fact, literature refers that most of the SODs are very stable in the range of temperatures from 25 to 45 °C, but SODs from thermophiles revealed higher stability [29–31]. The thermostability is often associated to the high number of charged residues, hydrophobic residues, increased number of ion-pairs, and increased buried surface [32, 33]. The number of charged residues (lysine, arginine, glutamic acid and aspartic acid, total 23.1 %) of ChrF is considerably higher than ChrC (total 17 %). This may explain in some ways the higher thermostability of ChrF.

It is well recognized that the toxic effect of chromate involves predominantly oxidative stress generated by the intracellular reduction of Cr(VI) to the highly reactive radical Cr(V) that through redox reactions ends as Cr(III) and results in ROS production [34, 35]. These species are directly implicated in damage of cellular components such as DNA and proteins [14, 36]. To assess the cellular oxidative stress generated by chromate in *O. tritici* cells and to evaluate a possible contribute of the ChrC or ChrF in ROS detoxification, the intracellular concentration of oxygen reactive species was measured using the specific probe H_2_DCFDA. Chromate treatment increased cytoplasmic ROS and this increase was clearly more visible in the SOD mutants. Previous studies with *E. coli* cells not challenged with chromate have already shown little degree of green fluorescence comparatively with the fluorescence signal from chromate-challenged cells [15]. In our results, an increase in the cytoplasmic ROS concentration also suggest that in *O. tritici* 5bvl1 the chromate induced more stress in ChrC and ChrF deficient cells.. Differences in SOD activities between the wild type and the mutants were also observed together with differences between cytoplasmic ROS levels. Comparing the ROS levels from both single mutants, we conclude that *chrC* mutation exerts a more drastic effect in cells even when SODs activities of both mutants were similar, which could be explained by the sensitivity of the techniques used. The differences observed in the ROS levels of the mutants could be related to the different predicted location of their SODs in cells. Cellular enzymes sharing similar functions, when subjected to stress conditions, could exhibit distinct performances. Studies of bacterial transcription analysis have shown that several genes known to be involved in response to oxidative stress were up-regulated under either chromate or dichromate stress. The up-regulation of *Sod* was observed for Cr(VI)-stressed *E. coli* [15], *Pseudomonas putida* [34] and *Caulobacter crescentus* [16] but not for *Shewanella oneidensis* MR-1 [37]. Moreover, *C. crescentus* showed differential expression of their SOD encoding genes under chromium stress. Its *SodA* showed an induction from 9-fold to 14-fold and the other two superoxide dismutase genes showed up to 2-fold induction [16].

The exploration of draft genome from strain *O. tritici* 5bvl1 showed two additional superoxide dismutase related genes which should code for functional SODs responsible for the general bacterial detoxification processes (unpublished results). These two identified SODs showed high homology between each other and they shared higher homology with ChrC than with ChrF. The presence of several SODs is a characteristic often present in bacteria. For instance in *Agrobacterium tumefaciens*, each SOD displays different expression pattern and cellular location [38].

## Conclusions

In summary, the chromate resistant strain *O. tritici* 5bvl1 carries a transposable element (Tn*Otchr*) that includes determinants coding for a chromate efflux pump and two distinct SOD enzymes. In addition to other putative SODs detected from bacterial genome analysis, the new characterized Fe-SOD (ChrC) and Mn-SOD (ChrF) ensure that superoxide anions are kept at physiologically safe levels allowing the growth of *O. tritici*. Moreover, the presence of ChrC and ChrF seems to be relevant to avoid ROS accumulation in chromate stressed cells.
